# Effects of light quality on growth, nutritional characteristics, and antioxidant properties of winter wheat seedlings (*Triticum aestivum* L.)

**DOI:** 10.3389/fpls.2022.978468

**Published:** 2022-09-02

**Authors:** Junyan Li, Xiaolei Guo, Siqi Zhang, Yinghua Zhang, Liping Chen, Wengang Zheng, Xuzhang Xue

**Affiliations:** ^1^National Research Center of Intelligent Equipment for Agriculture, Beijing, China; ^2^College of Agronomy and Biotechnology, China Agricultural University, Beijing, China

**Keywords:** wheat seedling, light quality, growth, nutritional characteristics, antioxidant property

## Abstract

Wheat seedlings are becoming popular for its high nutritional value. Effects of White (W), White + Red (WR), and White + Blue (WB) light-emitting diodes (LEDs) treatments on growth, nutritional characteristics and antioxidant properties of wheat seedlings were studied in a plant factory. The results showed that height, leaf area, shoot fresh, and shoot dry weight per wheat seedling were the highest under WR at 13 and 22 days after planting. Soluble sugar content in leaves and stems were 22.3 and 65% respectively higher under WB than those under W. Soluble protein content in leaves and stems were 36.8 and 15.2% respectively lower under WR than those under W. Contents of total flavonoids, glutathione (GSH) and ascorbic acid (ASA) in leaves were the highest under WB, whereas malondialdehyde (MDA) content in leaves was the lowest under WB. The activities of antioxidant enzymes [superoxide dismutase (SOD), peroxidase (POD), and ascorbate peroxidase (APX)] in leaves and 2,2-diphenyl-1-picrylhydrazyl (DPPH) radical scavenging ability were also the highest under WB. In conclusion, WR promoted the growth of wheat seedlings, and WB promoted antioxidant level and nutritional accumulation. This study provides guidance for wheat seedlings to carry out preferential production (biomass or quality).

## Introduction

Wheat (*Triticum aestivum* L.) is the most widely cultivated crop in the world, providing carbohydrates and proteins for human beings (Nazim Ud Dowla et al., 2018; Hu et al., 2021). Except for the grains, the seedlings of wheat, also named wheatgrass have been proved to be rich in flavonoids and polyphenols, which have the capability of scavenging reactive oxygen species (ROS) and are beneficial to human health (Durairaj et al., 2014; Bar-Sela et al., 2015; Jaiswal et al., 2018). Besides, wheat seedlings also contain proteins, sugars, triterpenes, and various antioxidant enzymes (Gawlik-Dziki et al., 2016; Ghumman et al., 2017; Kaur et al., 2021; Virdi et al., 2021). Adding wheat seedling juice to milk or meat can improve the nutritional value of the food (Devi et al., 2019). Wheat seedlings products are becoming widely used supplemental health foods (Kulkarni et al., 2006; Tsai et al., 2013; Choi et al., 2021).

Light is one of the most important variables affecting plant growth and development (Jiao et al., 2007; de Wit et al., 2016; Yadav et al., 2021). It not only provides energy source for photosynthesis, but also regulates seed germination, root architecture, shoot elongation, leaf expansion, circadian rhythms, phototropism, shade avoidance, flowering, chloroplast movement, and accumulation of phytochemical compounds such as phenolics (Bantis et al., 2018; Yadav et al., 2020). Modern agriculture has evolved towards the application of advanced technologies for plant cultivation in a controlled environment, exemplified with plant factories, in which light source is the most critical environment factor (Paradiso and Proietti, 2022). Light-emitting diodes (LEDs) are ideal light sources in plant factories, due to their advantages of small size, long service life, low energy consumption, low heat generation, and customized wavelengths compared with other artificial light sources (Lin et al., 2013; Bian et al., 2015; Ouzounis et al., 2015; Paradiso and Proietti, 2022). A white LED consists of a LED chip, which emits blue light with a narrow spectrum between 440 and 470 nm, and a coating of yellow phosphors. The light emitted by the phosphor, in combination with the remaining blue light leaking through the phosphor layer, result in a light which is perceived as white by the human eye (Gayral, 2017). White LEDs have been proven to maintain growth and development of plants (Singh et al., 2015; Guo et al., 2022a). However, the spectra of white LEDs are concentrated in the bluish visible light and other light qualities need to be added to meet the light needs of different plants at different growth stages (Cao et al., 2021).

Red and blue light account for the largest proportion of the total light received by plants (Nishio, 2000; Terashima et al., 2009). Proper combination of red and blue light can promote growth of *Oncidium*, perilla, lettuce, tomato, pepper, and *Lycoris longituba* (Liu et al., 2011; Goto et al., 2014; Zhang et al., 2018; Kaiser et al., 2019; Naznin et al., 2019; Li et al., 2022). Azad et al. (2020) demonstrated that red light promotes accumulation of carbohydrates in lettuce, while increasing blue light caused a decrease in fresh and dry weight in lettuce shoots (Son and Oh, 2013). It is worth noting that blue light has positive role in synthesis and accumulation of flavonoids and polyphenols (Lin et al., 2013; Zheng et al., 2019; Gao et al., 2021; Jung et al., 2021). In the study about *Salvia plebeian*, it has been found that the total phenolic and flavonoid contents were higher under red light supplemented with blue light (such as B:R = 3:7, B:R = 5:5, and B:R = 7:3) than in the monochromatic red light (Lee et al., 2020). Red and blue light induce changes of antioxidants level, including superoxide dismutase (SOD), peroxidase (POD), catalase (CAT), and ascorbate peroxidase (APX), ascorbic acid (ASA), and glutathione (GSH) (Mittler et al., 2004; You and Chan, 2015). Santos-Tierno et al. (2021) reported that red LEDs was effective in facilitating SOD and CAT activities in *Passiflora setacea*, while SOD and POD activities in ramie under red light were lower than those under white light (Rehman et al., 2020). According to Causin et al. (2006), blue light enhanced CAT activity in wheat compared with white light. Red and blue light lead to diverse morphological and physiological responses of plants, and these responses are species-specific. However, there are few studies on the roles of red and blue light in wheat seedlings, which is worth exploring.

In this experiment, three different LEDs light treatments (White, White + Red, and White + Blue) were designed to investigate the effects of red and blue light on the growth, nutritional quality and antioxidant capacity of wheat seedlings in a hydroponic system with the same light intensity and photoperiod in a plant factory. The results will provide some basic information for optimizing the spectral combinations of LEDs in production of wheat seedlings.

## Materials and methods

### Light treatments

**FIGURE 1:**
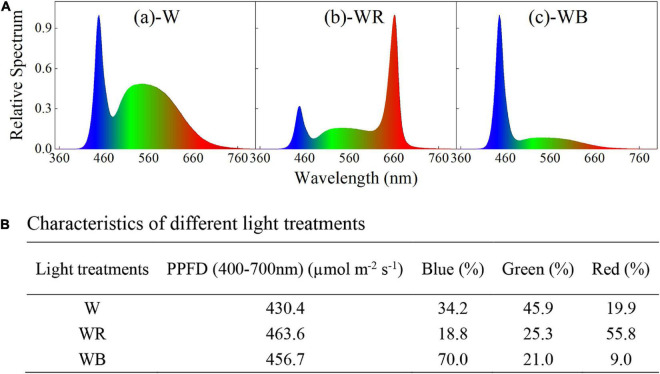
Spectral distribution **(A)** and characteristics **(B)** of different light treatments. **(A-a)** White light emitting diodes (LEDs) (W); **(A-b)** white plus red LEDs (WR); **(A-c)**, white plus blue LEDs (WB). Photosynthetic photon flux density (PPFD) for each light treatment was equal to 450 ± 20 μmol m^2^ s^−1^.

Three spectral combinations, white LEDs (W, B:G:R = 34:46:20, Figures 1A-a), white plus red LEDs (WR, B:G:R = 19:25:56, Figures 1A-b), and white plus blue LEDs (WB, B:G:R = 70:21:9, Figures 1A-c) were applied and wheat seedlings were planted in a plant factory for 43 days. The spectral characteristics of different light treatments were measured with a portable spectroradiometer (Plant Lighting Analyzer, V 2.00, China), and showed in Figure 1. Light intensity was kept constant at 450 ± 20 μmol m^–2^ s^–1^ throughout the experiment by moving up the light sources above the plants every week, where the photosynthetic photon flux density (PPFD) was measured with a photo/radiometer (Plant Lighting Analyzer, V 2.00, China). Lighting time per day was set to 12 h (12/12 h light/dark).

### Cultivation conditions

Winter wheat seeds (*T. aestivum* L., cv. ‘JiMai22’) were sterilized by 5% hydrogen peroxide (H_2_O_2_) for 30 min, then washed adequately 4 times in distilled water, and soaked in distilled water for 12 h. The seeds were imbibed in the dark on moistened germination paper at 25°C until plumule exposed 1 cm, and then vernalized for 15 days at 4°C. Before planting, germinated seeds grew for 3 days under white LEDs light (spectrum same as white LEDs, Figures 1A-a) for uniform growth (PPFD = 100 μmol m^–2^ s^–1^). The germinated seeds were planted into sponge cubes (2.0 cm × 2.0 cm × 2.0 cm) and hydroponically grown in a plant factory. Eighty-one plants spaced 8 cm apart were planted in a hydroponic box (80 cm × 80 cm × 10 cm). Each hydroponic box was used for one light treatment, and three hydroponic boxes as biological replicates in each light environment. During the experiment, wheat was grown for 43 days and randomly rotated every 5 days. A half-strength Hoagland’s solution was used, the pH of which was kept between 5.5 and 6.5, and renewed weekly (Hoagland and Arnon, 1950). Temperature during the experiment was maintained at 22 ± 1°C, and relative humidity was controlled at 65 ± 5%.

### Growth characteristics

Eight seedlings were taken from each treatment to measure plant height, leaf area and shoot dry weight at 13, 22, 31, and 40 days after planting (DAP). Plant height, length and width of all leaves of the samples were measured by a ruler, and the leaf area was calculated according to the coefficient method (Leaf area = Length × Width × 0.76) (Li et al., 2018; Guo et al., 2022b). Shoot fresh weight of the entire plant was determined by an electronic precision balance (CP224C, OHAUS, United States). After oven-dried at 105°C for 30 min, the samples were kept at 80°C for 72 h, and shoot dry weight was then measured by the electronic precision balance.

### Sampling

Fresh samples of 30 wheat seedlings (10 plants from the same hydroponic box as a biological repeat) were collected from each treatment at 43 DAP, and immediately frozen in liquid nitrogen, and then stored in a laboratory refrigerator at −80°C. The chemical used in the experiment were all supplied by Sigma-Aldrich (United States).

### Determination of soluble sugar and protein contents

Soluble sugar content was determined as described by Hanft and Jones (1986). Fresh sample (0.1 g) was ground in liquid nitrogen. After adding 3 ml deionized water, the grounded sample was heated in a boiling water bath for 20 min, centrifuged at 4,500 *g* for 10 min, and the supernatant was taken. A total of 3 ml deionized water was added to the residue, extracted twice repeatedly. After combining the resulting supernatants, deionized water was added to adjust the volume to 10 ml. Extract solution (1 ml) was mixed with 4 ml anthrone solution (2 g L^–1^), and then soaked in a water bath at 45°C for 12 min. After cooling in the dark, the absorbance of the mixture was detected at 625 nm by a microplate reader (Multiskan FC, Thermo Scientific™, United States), and soluble sugar content was calculated using a sucrose standard curve with concentrations ranging from 0 to 2 mg ml^–1^ (*R*^2^ = 0.9997).

Soluble protein content was measured by the Coomassie brilliant blue colorimetry method (Bradford, 1976). A total of 0.1 g fresh sample was homogenized in 2 ml distilled water. The mixture was centrifuged at 3,000 *g* for 10 min, and 1 ml supernatant was mixed with 5 ml Coomassie brilliant blue G-250 solution (0.1 g L^–1^). After 2 min, the absorbance of the mixture was detected at 595 nm by the microplate reader, and soluble protein content was calculated from standard curves (*R*^2^ = 0.9968).

### Determinations of malondialdehyde, glutathione, and ascorbic acid contents

Fresh sample (0.2 g) was ground into homogenate with 5 ml trichloroacetic acid solution (TBA, 5%, w/v), and centrifuged at 15,000 *g* for 15 min. The supernatant was used for measurement of malondialdehyde (MDA) and GSH contents.

The method described by Hameed et al. (2021) was used to quantify MDA content, with slight modification. 1 ml supernatant was mixed with 1 ml TBA solution (0.67%, w/v), incubated in boiling water for 30 min and then quickly cooled in an ice-bath. The mixture was centrifuged and the absorbance was detected at 450, 532, and 600 nm. MDA content was determined using the following equation:


(1) $$\mathrm{MDA}\,\left(\mu \,\text{mol}\,{\text{g}}^{-1}\,\mathrm{FW}\right)=\frac{\left(6.45\times \left(\mathrm{OD532}-\mathrm{OD600}\right)-0.56\times \mathrm{OD450}\right)\times \text{V}}{1000\times \text{W}}$$

where V is the total volume of supernatant and W is the fresh weight of the sample.

Glutathione content was measured according to the method by Qiu et al. (2014) with slight modification. A total of 2 ml supernatant was added to 4 ml phosphate buffer (200 mM, pH 7.0) and 0.4 ml 5, 5-dithiobis (2-nitrobenzoic) solution (0.396 mg ml^–1^, pH 7.0). After 5 min at 30°C, the absorbance was determined at 412 nm by the microplate reader and GSH content was calculated based on the GSH standard curve with concentrations ranging from 0 to 80 μg ml^–1^ (*R*^2^ = 0.9976).

Ascorbic acid content was determined according to the method by He et al. (2020). Fresh sample (0.1 g) was extracted with 1 ml oxalic acid EDTA solution (1%, w/v), and centrifuged at 4,000 *g* for 10 min. 0.2 ml supernatant was mixed with 0.8 ml oxalic acid EDTA solution (1%, w/v), 0.1 ml phosphate-acetic acid solution (3%, w/v), 0.2 ml vitriol (5%, v/v), and 0.4 ml ammonium molybdate solution (5%, w/v). After 15 min, the absorbance of mixture solution was measured at 705 nm by the microplate reader, and ASA content was calculated based on the standard curve with concentration ranging from 0 to 1 mg ml^–1^ (*R*^2^ = 0.9977).

### Determination of total flavonoids, triterpenes, and polyphenols contents

Fresh sample (1.0 g) was ground in liquid nitrogen, and extracted with 25 ml methanol solution (80%, v/v) ultrasonically for 20 min, and centrifuged to obtain supernatant. The above process was repeated three times, and all supernatants were pooled and volume was adjusted to 100 ml with methanol solution (80%, v/v). The methanol extract was stored at −20°C for determination of the contents of flavonoids, triterpenes, and polyphenols, and the free radical scavenging abilities of 2,2′-azino-bis (3-ethylbenzthiazoline-6-sulfonic acid) (ABTS) and 2,2-diphenyl-1-picrylhydrazyl (DPPH).

The total flavonoids content was measured using the method described by Guo et al. (2013). A total of 2 ml extract, 0.2 ml NaNO_2_ solution (5%, w/v), 0.2 ml Al(NO_3_)_3_ solution (10%, w/v), and 2 ml NaOH solution (1 M) were mixed adequately. The absorbance of the mixture was measured at 510 nm by the microplate reader after standing for 15 min. Total flavonoids content was expressed as milligram rutin equivalent gram^–1^ in fresh weight (mg RE g^–1^ FW) through the calibration curve of rutin with the concentration ranging from 0 to 20 μg ml^–1^ (*R*^2^ = 0.9974).

The total triterpenes content was measured by the method of Jiang et al. (2021). A total of 100 μl extract were mixed with 100 μl vanillin-acetic acid solution (2.5%, w/v) and 200 μl perchloric acid solution. After incubation at 60°C for 15 min, 650 μl glacial acetic acid was added and adequately mixed. The absorbance of mixture was detected at 550 nm by the microplate reader after standing for 10 min. The total triterpenes content was expressed as milligram ursolic acid equivalent gram^–1^ in fresh weight (mg UAE g^–1^ FW) and calculated from the standard curve (concentration ranging from 20 to 120 μg ml^–1^, *R*^2^ = 0.9918).

The total polyphenols content was measured using the method described by Sarker and Oba (2020). A total of 100 μl extract was mixed with the Folin–Ciocalteu reagent (2 N, 50 μl). A total of 400 μl Na_2_CO_3_ solution (1 M) and 1 ml deionized water were added after 5 min. The mixture was incubated at 37°C for 90 min and the absorbance was measured at 740 nm by the microplate reader. The content of the total polyphenols was expressed as gallic acid equivalent standard of fresh weight (mg GAE g^–1^ FW) through the calibration curve of gallic acid with concentration ranging from 0 to 50 μg ml^–1^ (*R*^2^ = 0.9983).

### Determination of antioxidant activities

ABTS radical scavenging ability was measured using the method by Jiang et al. (2021). Equal volume of 7.4 mM ABTS solution and 2.6 mM K_2_S_2_O_8_ solution were mixed at room temperature in the dark for 20 h to generate ABTS+ stock solution. A total of 5 mM PBS solution (pH 7.4) was used to dilute ABTS+ stock solution to absorbance of 0.75 at 734 nm to prepare working solution. A total of 200 μl of working solution was mixed with 40 μl extract. After incubation in the dark for 6 min, the absorbance was read at 734 nm by the microplate reader. The ABTS radical scavenging ability was calculated from the Trolox calibration curve (concentration ranging from 0 to 0.05 mg ml^–1^, *R*^2^ = 0.999) and expressed as Trolox equivalent standard of fresh weight (mg TE g^–1^ FW).

2,2-Diphenyl-1-picrylhydrazyl radical scavenging ability was also measured using the method by Jiang et al. (2021) with small adjustment. A total of 200 μl DPPH solution (0.15 mM) was blended with 100 μl extract in a 96-well-plate. After incubation at 37°C for 30 min, the absorbance was read at 517 nm by the microplate reader. The result was expressed as ASA equivalent per gram of fresh weight (mg AE g^–1^ FW) based on the standard curve (concentration ranging from 0.005 to 0.4 mM, *R*^2^ = 0.9976).

### Antioxidant enzymes activities

Fresh sample (0.5 g) was ground in liquid nitrogen, and extracted into a homogenate with 3 ml of ice-cold extraction buffer, which contained phosphate buffer (50 mM, pH 7.8) and EDTA (1 mM). The homogenate was centrifuged at 15,000 *g* for 15 min at 4°C, and the supernatant was used to measure the activities of SOD, POD, CAT, and APX.

Superoxide dismutase activity was measured based on the method proposed by Giannopolitis and Ries (1977), with slight modifications. A total of 3 ml reaction mixture was composed of 0.1 ml supernatant, phosphate buffer (50 mM, pH 7.8), EDTA (0.1 mM), methionine (130 mM), nitroblue tetrazolium (NBT, 0.4 mM), and riboflavin (0.02 mM). The reaction was started by placing the reaction tube under two 40 W fluorescent lamps and stopped after 20 min by removing the reaction tube from the light source. Light reactions with no light and no supernatant were used as calibration standards. The absorbance of the reaction mixture was read at 560 nm. One unit of SOD activity (U) was defined as the amount of enzyme required to produce 50% inhibition of NBT reduction.

Peroxidase activity was measured based on the method of Qiu et al. (2011) with slight changes. Added 0.1 ml supernatant to a mixture consisting of phosphate buffer (50 mM, pH 7.0), guaiacol (0.05%, w/v), EDTA (0.1 mM), and H_2_O_2_ (2%, v/v). By tracking the increase in absorbance at 470 nm over 5 min and quantified by the amount of tetraguaiacol formed using its molar extinction coefficient (26.6 mM^–1^ cm^–1^). One unit of POD activity (U) was defined as a decrease in absorbance value of 0.01 per minute.

Catalase activity was measured with the method of Cakmak and Marschner (1992). A total of 0.1 ml supernatant was added to a mixture of phosphate buffer (100 mM, pH 7.0), EDTA (0.1 μM), and H_2_O_2_ (0.1%, v/v). The reaction was measured by tracking the decrease in absorbance at 240 nm for 3 min and quantified by its molar extinction coefficient (39.4 mM^–1^ cm^–1^). One unit of CAT activity (U) was defined as a decrease in absorbance value of 0.01 per minute.

Ascorbate peroxidase activity was measured with the method of Nakano and Asada (1981). A total of 3 ml reaction solution consisted of 0.1 ml supernatant, phosphate buffer (50 mM, pH 7.8), EDTA (0.1 mM), ascorbate (0.5 mM), and H_2_O_2_ (0.1 mM). The resulting mixture was quantified by tracking the increase in absorbance at 290 nm for 1 min and by its molar extinction coefficient (2.8 mM^–1^ cm^–1^). One unit of APX activity (U) was defined as an increase in absorbance value of 0.01 per minute.

### Statistical analysis

The data were subjected to one-way ANOVA using SPSS statistical software (PASW statistical version 26.0, SPSS Inc., Chicago, IL, United States). Duncan’s new multiple range test was applied to identify differences between means at the ^∗^*P* < 0.05, ^∗∗^*P* < 0.01, and ^∗∗∗^*P* < 0.001 level. Correlations between growth, quality characteristics, antioxidant properties of wheat seedlings and light environments were uncovered through Pearson correlation analysis based on the percentage composition of light quality in the treatments (Figure 1B). All figures were created using OriginPro 2022 (OriginLab Corp., United States) or Adobe Illustrator CC 242 2019 (Adobe Inc., United States).

## Results

### Growth characteristics

**FIGURE 2:**
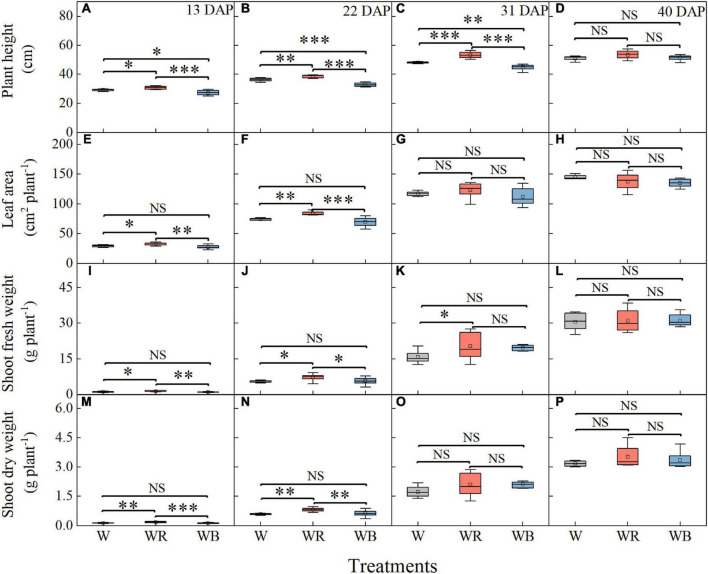
Plant height **(A–D)**, leaf area **(E–H)**, shoot fresh weight **(I–L)**, and shoot dry weight **(M–P)** of wheat seedlings under different treatments at 13, 22, 31, and 40 days after planting (DAP). Values represent the mean ± SE (*n* = 8). The symbols ^∗^, ^∗∗^, and ^∗∗∗^ indicate significance at the 0.05, 0.01, and 0.001 levels, respectively. NS, not significant, according to the Duncan’s test.

As showed in Figure 2, growth of wheat seedlings was significantly influenced by light qualities. The highest plant height of wheat seedlings was observed under WR and lowest was observed under WB at 13, 22, and 31 DAP (Figures 2A–C). Leaf area, shoot fresh and dry weight per wheat seedling was highest under WR at 13 and 22 DAP (Figures 2E,F,I,J,M,N). Shoot fresh weight was higher under WR than that under W at 31 DAP (Figure 2K). Difference in plant height and shoot fresh weight among the three light treatments disappeared at 40 DAP (Figures 2D,L), and difference in leaf area and shoot dry weight disappeared at 31 and 40 DAP (Figures 2G,H,O,P).

### Soluble sugar and soluble protein

**FIGURE 3:**
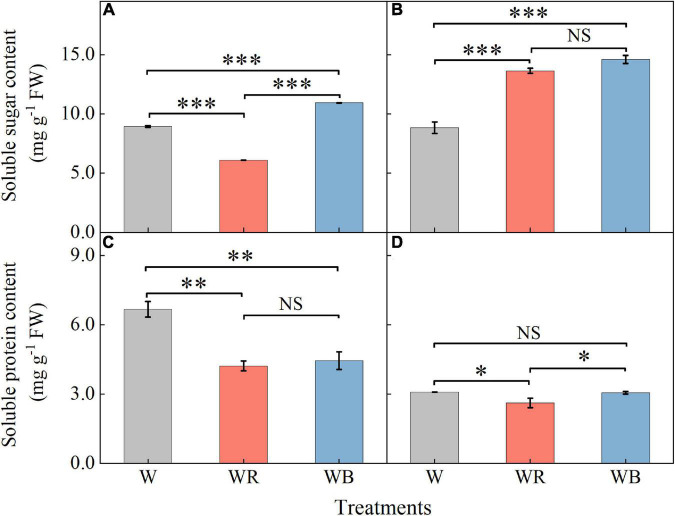
The contents of soluble sugar and soluble protein in extracts of wheat seedlings leaves **(A,C)** and stems **(B,D)** under different treatments. Values represent the mean ± SE (*n* = 3). The symbols ^∗^, ^∗∗^ and ^∗∗∗^ indicate significance at the 0.05, 0.01, and 0.001 levels, respectively. NS, not significant, according to the Duncan’s test.

The contents of soluble sugar and soluble protein in wheat seedling leaves and stems were shown in Figure 3. Soluble sugar contents under WB increased 22.3% in leaves and 65.0% in stems compared with those under W (Figures 3A,B). Soluble protein content under WB decreased 33.5% in leaves than that under W (Figure 3C). Soluble sugar contents under WR increased 54.1% in stems (Figure 3B), and decreased 31.8% in leaves compared than those under W (Figure 3A). Soluble protein contents were 36.8% lower in leaves and 15.2% lower in stems than those under W (Figures 3C,D).

### Total flavonoids, triterpenes, and polyphenols

**FIGURE 4:**
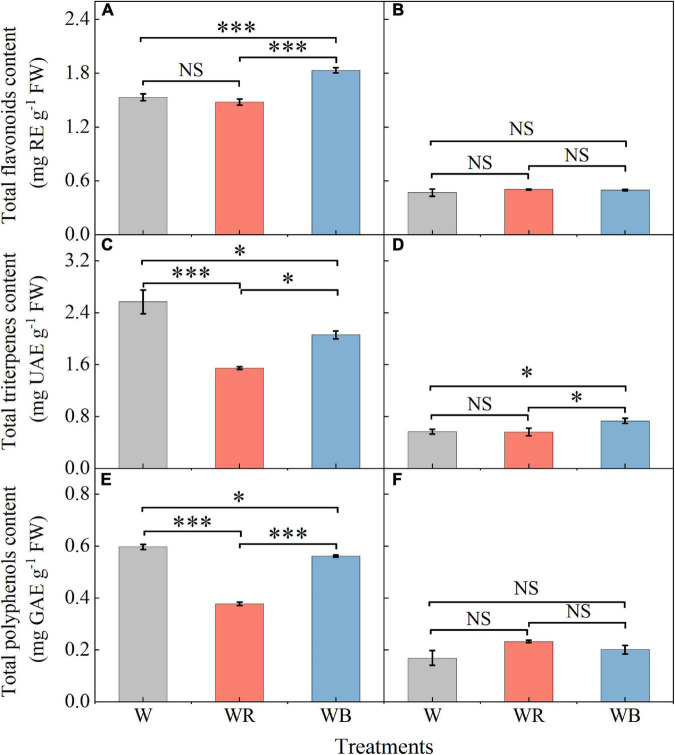
The contents of total flavonoids, triterpenes and polyphenols in extracts of wheat seedlings leaves **(A,C,E)** and stems **(B,D,F)** under different treatments. Values represent the mean ± SE (*n* = 3). The symbols ^∗^ and ^∗∗∗^ indicate significance at the 0.05 and 0.001 levels, respectively. NS, not significant, according to the Duncan’s test.

The contents of total flavonoids, triterpenes and polyphenols in leaves and stems of wheat seedlings grown under different LEDs treatments were provided in Figure 4. Compared with W, WB enhanced total flavonoids content in leaves by 19.3% (Figure 4A), and total triterpenes content in stems by 29.4% (Figure 4D). Compared with W, WR, and WB resulted in a significant decrease in total triterpenes and polyphenols content in leaves (Figures 4C,E). There was no significant difference in the contents of total flavonoids and polyphenols in stems among all treatments (Figures 4B,F).

### Lipid peroxidation and antioxidants

**FIGURE 5:**
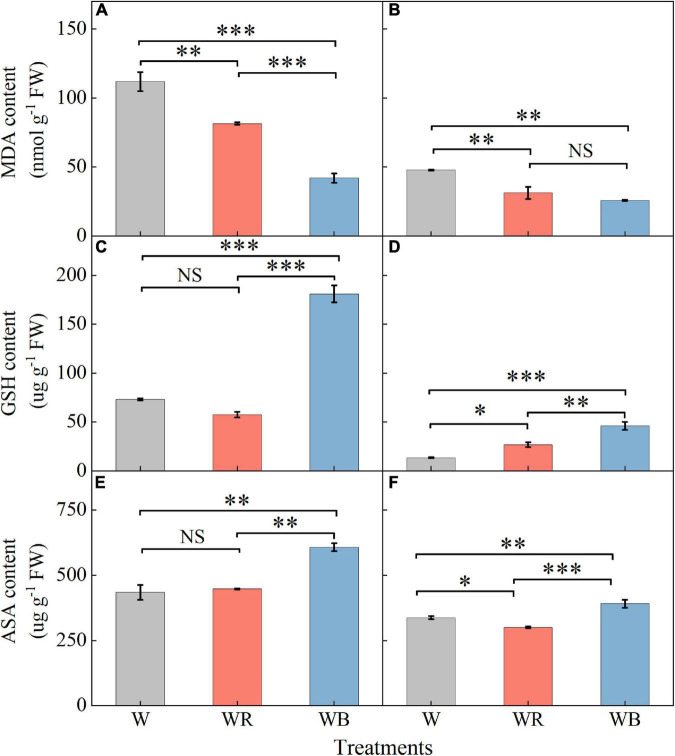
The contents of malondialdehyde, glutathione and ascorbic acid in extracts of wheat seedlings leaves **(A,C,E)** and stems **(B,D,F)** under different treatments. Values represent the mean ± SE (*n* = 3). The symbols ^∗^, ^∗∗^ and ^∗∗∗^ indicate significance at the 0.05, 0.01, and 0.001 levels, respectively. NS, not significant, according to the Duncan’s test.

Contents of MDA, GSH, and ASA in leaves were higher than those in stems (Figure 5). MDA contents of leaves and stems under WB and WR were significantly lower than those under W (Figures 5A,B). Contents of GSH and ASA in leaves and stems were the highest under WB (Figures 5C–F). GSH content in stems increased 98.6% under WR than that under W (Figure 5D). ASA content in stems decreased 11.0% under WR than that under W (Figure 5F). Contents of GSH and ASA in leaves under WR and W were not significantly different (Figures 5C,E).

### Antioxidant enzymes

**FIGURE 6:**
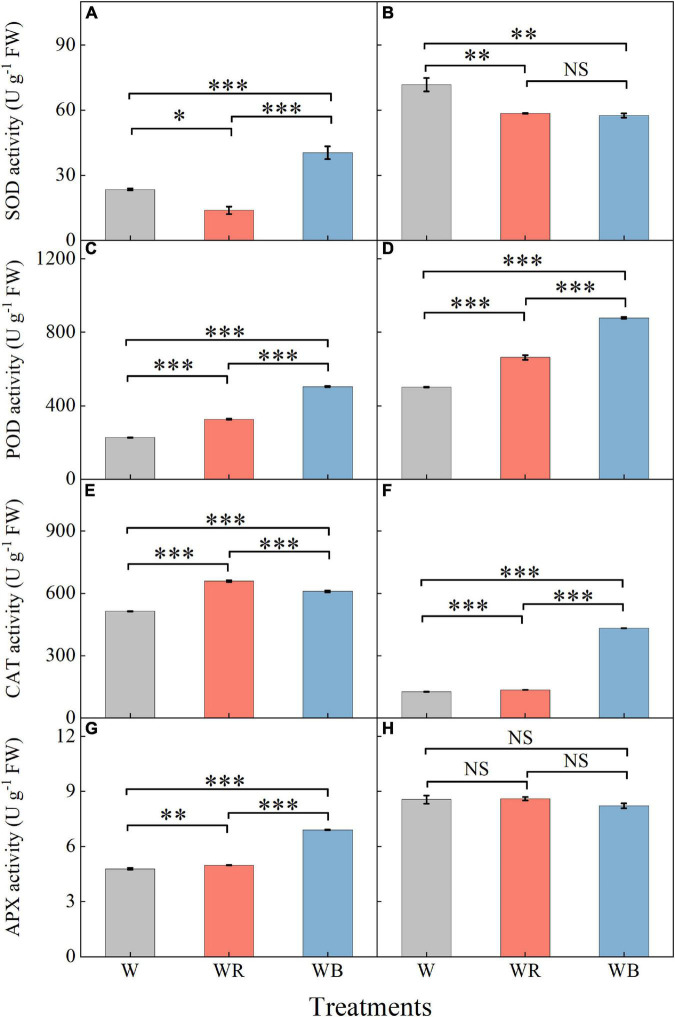
The activities of SOD, POD, CAT, and APX in extracts of wheat seedlings leaves **(A,C,E,G)** and stems **(B,D,F,H)** under different treatments. Values represent the mean ± SE (*n* = 3). The symbols ^∗^, ^∗∗^ and ^∗∗∗^ indicate significance at the 0.05, 0.01, and 0.001 levels, respectively. NS, not significant, according to the Duncan’s test.

The activities of antioxidant enzymes in leaves and stems were affected by light qualities (Figure 6). Compared with W, WB increased SOD activity of leaves by 72.1% (Figure 6A), and decreased SOD activity of stems by 19.8% (Figure 6B). SOD activity under WR decreased 40.9% in leaves and 18.3% in stems compared with those under W (Figures 6A,B). POD and CAT activities in leaves and stems under WR and WB were significantly higher than those under W (Figures 6C–F). APX activities in leaves under WR and WB were higher than that under W (Figure 6G). No significant difference in stem APX activity was observed among the three treatments (Figure 6H).

### Antioxidant capacity

**FIGURE 7:**
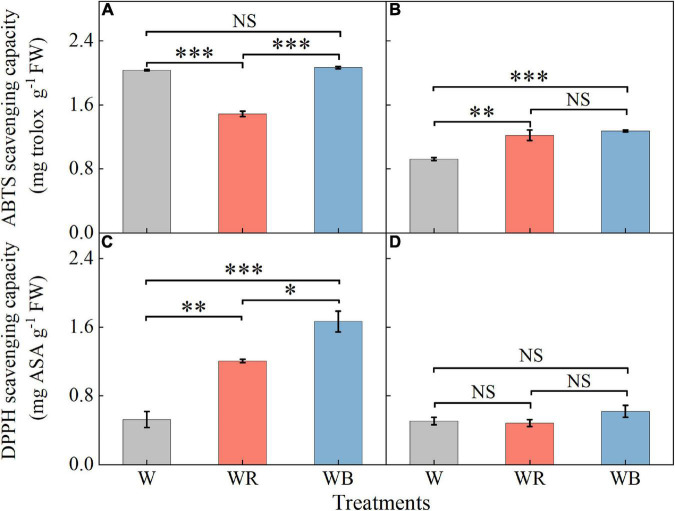
The values of ABTS and DPPH scavenging capacity in extracts of wheat seedlings leaves **(A,C)** and stems **(B,D)** under different treatments. Values represent the mean ± SE (*n* = 3). The symbols ^∗^, ^∗∗^ and ^∗∗∗^ indicate significance at the 0.05, 0.01, and 0.001 levels, respectively. NS, not significant, according to the Duncan’s test.

The ABTS and DPPH radical scavenging capacities in leaves were higher than those in stems (Figure 7). Compared with W, WR reduced ABTS radical scavenging capacity in leaves by 26.7% (Figure 7A). Compared with W, WB, and WR increased ABTS radical scavenging capacity in stems by 32.1 and 37.9%, respectively (Figure 7B), and DPPH radical scavenging ability in leaves by 130.6 and 218.5%, respectively (Figure 7C). There was no significant difference in DPPH radical scavenging ability in stems among all the three treatments (Figure 7D).

### Comprehensive effects of light qualities on plant growth, nutrition, and antioxidant properties

**FIGURE 8:**
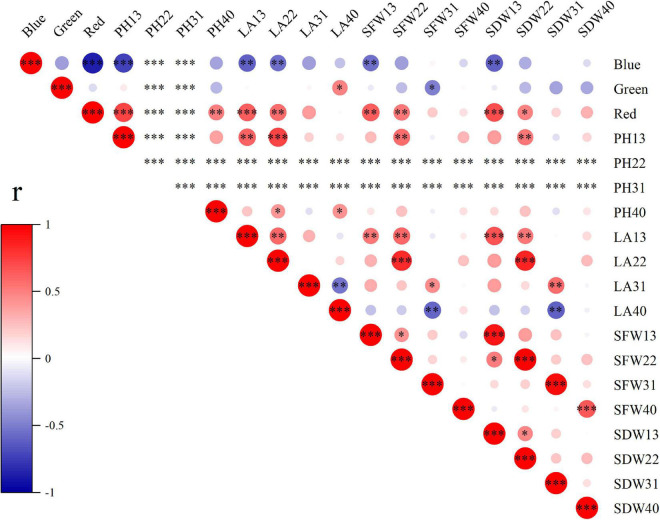
The correlation among light spectral quality (blue, green, and red) and growth indicators. PH, plant height; LA, leaf area; SFW, shoot fresh weight; SDW, shoot dry weight. Difference test at the ^∗^*P* < 0.05, ^∗∗^*P* < 0.01, and ^∗∗∗^*P* < 0.001 level. The colors reflect the changes in correlation coefficient: red color represents correlation coefficient with high and positive correlation and blue indicates high and negative correlation.

**FIGURE 9:**
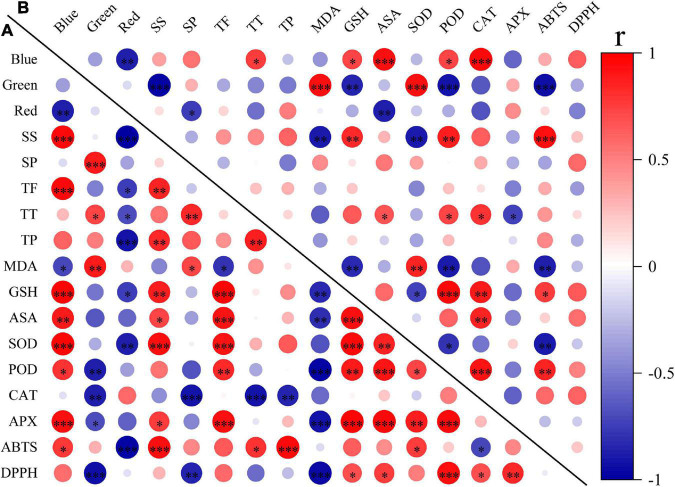
The correlation among light spectral quality (blue, green, and red) and nutritional and antioxidant indicators in leaves **(A)** and in stems **(B)**. SS, soluble sugar content; SP, soluble protein content; TF, total flavonoids; TT, total triterpenes; TP, total polyphenols; MDA, malondialdehyde; GSH, glutathione; ASA, ascorbic acid; SOD, superoxide dismutase; POD, peroxidase; CAT, catalase; APX, ascorbate peroxidase; ABTS, 2,2′-azino-bis (3-ethylbenzthiazoline-6-sulfonic acid); DPPH, 2,2-diphenyl-1-picrylhydrazyl. Difference test at the ^∗^*P* < 0.05, ^∗∗^*P* < 0.01, and ^∗∗∗^*P* < 0.001 level. The colors reflect the changes in correlation coefficient: red color represents correlation coefficient with high and positive correlation and blue indicates high and negative correlation.

To explore the effects of light spectral qualities (blue, green, and red) of light treatments, correlation between light spectral qualities and growth characteristics (Figure 8), nutritional and antioxidant characteristics (Figure 9) of wheat seedlings was analyzed. Among the measured parameters of growth characteristics, blue light was significantly negatively correlated with most parameters, and red light was significantly positively correlated with most parameters (Figure 8). The situation was different when it came to parameters in terms of nutritional characteristics and antioxidant properties (Figure 9). In leaves, blue light was significantly positively correlated with SS (soluble sugar), TF (total flavonoids), GSH, ASA, SOD, POD, APX, and ABTS, and significantly negatively correlated with MDA. In stems, blue light was significantly positively correlated with TT (total triterpenes), GSH, ASA, POD, and CAT. Conversely, red light was significantly negatively correlated with SS, TF, TT, TP (total polyphenols), GSH, SOD, and ABTS in leaves, and was significantly negatively correlated with SP (soluble protein) and ASA in stems. In addition, there was a significant positive correlation among TF, GSH, ASA, SOD, POD, and APX in leaves. MDA was significantly negatively correlated with TF, GSH, ASA, POD, APX, and DPPH.

## Discussion

Growth and development of plants are affected by wavelengths of light, among which red and blue light are regarded as the most influential wavelengths, attracting much attention (Zheng et al., 2019; Li et al., 2020; Jung et al., 2021). Our results showed that red light promoted the growth of wheat seedlings, mainly manifested as increased plant height, leaf area, shoot fresh, and dry weight per plant under WR (B:G:R = 19:25:56) (Figure 2). Similar results have been reported in water spinach by Kitayama et al. (2019) that red LEDs light resulted in a high level of stem fresh weight and plant height. WB (B:G:R = 70:21:9) had a negative effect on wheat growth (Figure 2), which is similar to the result of Wollaeger and Runkle (2015). That might be because high proportion of blue light under WB inhibited stem elongation and leaf area expansion (Kalaitzoglou et al., 2021). Interestingly, the effect of red and blue light on growth of wheat seedlings diminished over time (Figure 8), which indicated that this effect might be related to the growth stage of wheat or an acclimation response to the new light conditions.

Soluble sugars, soluble proteins, flavonoids, triterpenoids, and polyphenols are important primary and secondary metabolites of plants, which have been proved to be beneficial for human health (Augustin et al., 2011; Kumar and Pandey, 2013; Jakobek, 2015; Rienth et al., 2021). It has been reported that light quality is an important factor affecting the synthesis and accumulation of these metabolites (Jeong et al., 2012; Bae et al., 2016; He et al., 2020; Jiang et al., 2021). In our study, the contents of soluble sugar and total flavonoids content in leaves were highest under WB (B:G:R = 70:21:9), and the contents of soluble protein, total triterpenoids, and polyphenols was highest under W (B:G:R = 34:46:20) (Figures 3, 4), which is similar to the results of the study by Son and Oh (2013). However, Zhang et al. (2018) reported different results in lettuce. They found that soluble sugar content was the highest under red light and 1B:9R, while soluble protein content was the highest under blue light and 1B:4R. Different results may be related to species. Blue light was positively correlated with TF in leaves (Figure 9A), which may be because blue light enhances the activities of general phenylpropanoid and flavonoid pathways enzymes, thereby promoting the synthesis and accumulation of total flavonoids in leaves (Koyama et al., 2012; Zoratti et al., 2014).

Abiotic stress can break the balance of ROS production and elimination, resulting in excessive accumulation of ROS, and ultimately oxidative stress (Gill and Tuteja, 2010). MDA is the oxidation end product of lipid peroxidation caused by free radicals, which can indirectly reflect the degree of oxidative stress (Saleem et al., 2019). In our study, WB (B:G:R = 70:21:9) decreased MDA content in leaves and stems of wheat seedlings compared with W (B:G:R = 34:46:20) (Figure 5). Similar results have been reported that MDA content of wheatgrass under 75B:25R was lower in comparison with 18B:64G:18R (Bartucca et al., 2020).

Plants are well equipped with a variety of antioxidants to scavenge oxidative stress (Gill and Tuteja, 2010). Among them, GSH and ASA are important non-enzymatic antioxidant, which donate electrons for antioxidant enzymes to scavenge ROS (Noctor and Foyer, 1998; Akram et al., 2017; Zhitkovich, 2021). SOD, POD, CAT, and APX are crucial enzymatic antioxidants that cooperate to detoxify excessive ROS (You and Chan, 2015). Numerous studies have shown that the responses of antioxidants vary with the proportion of red and blue light, plant species, their genotypes and development stages (Choi et al., 2013; Manivannan et al., 2017; Ye et al., 2017; Yu et al., 2017; Liu et al., 2018). In our study, WB (B:G:R = 70:21:9) increased the contents of GSH and ASA (Figure 5), which may be due to blue light changes transcription signal transduction and metabolism of GSH and ASA metabolism (Liu and Zhang, 2021). WB also enhanced activities of antioxidant enzymes (SOD, POD, CAT, and APX) in leaves or stems of wheat seedlings (Figure 6), which is similar to the results reported by Kook et al. (2013), who found blue LEDs light improved CAT and APX activities of lettuce. Non-enzymatic antioxidants (ASA and GSH) were positively correlated with enzymatic antioxidants (SOD, POD, and APX) in leaves (Figure 9A), which is related to their synergistic cooperation in scavenging ROS (Mittler et al., 2004; Hasanuzzaman et al., 2020). In addition, TF was also positively correlated with these antioxidants (ASA, GSH, SOD, POD, and APX) in leaves (Figure 9A), which might be the antioxidant-induced change in cellular redox homeostasis that activates the biosynthesis of flavonoids (Agati et al., 2012).

ABTS and DPPH radical scavenging capacity are two commonly used parameters to indicate the total antioxidant capacity of plants (Floegel et al., 2011). WB could effectively improve the ABTS radical scavenging capacity in stems and DPPH radical scavenging capacity in leaves. Apparently, occurrence of higher contents of non-enzymatic antioxidants (ASA and GSH) and higher activities of antioxidant enzymes (SOD, POD, CAT, and APX) under the WB positively affected the ABTS and DPPH radical scavenging capacities in leaves and stems extract. Similar results were obtained in *Rehmannia glutinosa* (Manivannan et al., 2015) and *P. setacea* (Santos-Tierno et al., 2021). Consequently, our results suggest that WB (B:G:R = 70:21:9) have the capacity to enhance antioxidant defense mechanism and to elicit accumulation of potential secondary metabolites in wheat seedlings leaves.

## Conclusion

This study investigated growth, nutrition, and antioxidant characteristics of wheat seedlings under LEDs sources with different spectra combinations. Plant height, leaf area, shoot fresh, and dry weight were the highest under WR at the early stage of planting. Contents of soluble sugar, soluble protein, triterpenoids, polyphenols, flavonoids, ASA, and GSH were higher and MDA content was lower under WB than those under W. Activities of antioxidant enzymes (SOD, POD, CAT, and APX) and ABTS and DPPH radical scavenging capacities in leaves or stems under WB were higher than those under W. LEDs with a high ratio of red light promoted growth of wheat seedlings, and LEDs with a high ratio of blue light reduced damage of membrane lipid peroxidation and improved antioxidant capacity by increasing activities of antioxidant enzymes and contents of antioxidants. Our study provides a basis for selecting appropriate ratio of red to blue according to the production aim.

## Data availability statement

The raw data supporting the conclusions of this article will be made available by the authors, without undue reservation.

## Author contributions

JL and XG performed the experiment, analyzed the data, and wrote the manuscript. SZ participated in the experiment. XX and YZ designed and supervised the research. LC and WZ reviewed and edited the manuscript. All authors have read and agreed to the published version of the manuscript.
